# GC-MS Characterization of Volatile Flavor Compounds in Stinky Tofu Brine by Optimization of Headspace Solid-Phase Microextraction Conditions

**DOI:** 10.3390/molecules23123155

**Published:** 2018-11-30

**Authors:** Hui Tang, Jin-Kui Ma, Lin Chen, Li-Wen Jiang, Jing Xie, Pao Li, Jing He

**Affiliations:** 1 College of Food Science and Technology, Hunan Agricultural University, Changsha 410128, China; lokeytang@163.com (H.T.); xieliangsicq@163.com (J.X.); lipao@mail.nankai.edu.cn (P.L.); m15216896160_1@163.com (J.H.); 2 Henry Fok School of Food Science and Engineering, Shaoguan University, Shaoguan 51200, China; jscl2009@163.com; 3 School of Food & Pharmaceutical Engineering, Zhaoqing University, Zhaoqing 526061, China; jinkuima@gmail.com; 4 Hunan Provincial Key Laboratory of Food Science and Biotechnology, Changsha 410128, China

**Keywords:** HS-SPME-GC-MS, optimum condition, stinky tofu, brine, volatile flavor compounds, principal component analysis

## Abstract

This study optimized the headspace solid phase microextraction (HS-SPME) conditions for the analysis of the volatile flavor compounds of Chinese south stinky tofu brine by gas chromatography-mass spectrometry (GC-MS). The optimum HS-SPME conditions established were as follows: polar column CD-WAX, white 85 μm polyella extractor, extraction temperature 60 °C, equilibrium time 20 min, extraction time 40 min. Under these conditions, a total of 63 volatile flavor compounds in five stinky tofu brines were identified. The offensive odor of the stinky tofu may be derived from some of the volatile flavor compounds such as phenol, p-cresol, 3-methylindole, indole, acetic acid, propionic acid, isobutyric acid, n-butyric acid and 3-methylbutanoic acid. The volatile flavor substances data was examined by principal component analysis (PCA) to visualize the response patterns in the feature space of principal components (PC). PCA analysis results revealed that the Chengshifu brine (STB1) and Baise jingdian brine (STB4) are similar in PC 1, 2, and 3, and the two brines have a similar flavor. Results also indicate that the Huogongdian brine (STB2) and Wangcheng brine (STB3) can be grouped in the same class as they are similar in PC 3. However, PC 1, 2, and 3 of the Luojia brine (STB5) and other brands of brine are different as is the flavor.

## 1. Introduction

Stinky tofu, known as Oriental cheese, is one of the time-honored traditional snacks in China. According to the process technology, there are two kinds of stinky tofu: southern stinky tofu and northern stinky tofu. Changsha stinky tofu, as one of the famous southern stinky tofu, is a consumer favorite for its characteristics of being “black as ink,” “fragrant as alcohol,” “tender as crisp,” “soft as velvet” and “rich in nutrition” [1,2,3,4]. Changsha stinky tofu, a delicious food, is first dipped into the fermented brine for several hours or days. After this, it is subject to deep-frying before being mixed with seasonings. Stinky tofu brine is the key point in the process, which is made by a natural fermentation of soy sauce, mushroom, amaranth, and bamboo shoots mixed with water from several months to 1–2 years [5]. The brine is not directly edible. However, the transfer of microorganisms, functional components, and flavor substances occurring in the process of marinating tofu in brine, gives it a special flavor of “smells bad but tastes good.”

The recognition of the importance of the volatile flavor compounds for stinky tofu was relatively late. In an early study, Qi investigated the volatile flavor substances of non-fermented stinky tofu and found that isovaleraldehyde, *trans*-2,*trans*-4-decadien-1-al, dimethyl disulfide, p-cresol, ethyl butyrate, 1-octen-3-ol, and 1-nonanal are important volatile flavor components for the unfermented stinky tofu. These volatile flavor components are mainly derived from the degradation of free amino acids and fatty acids in soy products, natural fruits, and plants [6]. In recent years, several research projects were conducted on the flavor substances of stinky tofu and brine. Liu optimized the extraction conditions of SPME and studied the organic volatile flavor compounds in the fermentation of Beijing Wangzhihe stinky tofu (northern stinky tofu) by GC-MS method. A total of 39 volatile components were identified in this work including indole, dimethyl trisulfide, phenol, dimethyl disulfide, and dimethyl tetrasulfide [7]. Sun analyzed the species and content of characteristic aroma components in Beijing Wangzhihe stinky tofu using SDE (simultaneous distillation extraction) combined with GC-MS identified 44 volatile components, in which the indole and sulfur compounds are mainly derived from the further degradation of free amino acids and sulfur-containing amino acids [8]. However, none of these studies involved southern stinky tofu brine. Zheng compared the volatile components in both dark color and light color stinky tofu brine by the SPME-GC-MS method. Results showed that there are 38 kinds of volatile flavor substances in the dark-colored stinky tofu and 42 kinds of volatile flavor substances in the light-colored stinky tofu. Both stinky tofu brines share nine kinds of volatile components [9]. Liu compared the difference in volatile substances extracted by four different extraction fibers by using the HS-SPME method with fermented stinky tofu as raw material [7]. However, these researches did not systematically optimize the analysis conditions and methods for the flavor substances. Moreover, due to the wide variety of stinky tofu, the differences in flavor substances between different brands of southern stinky tofu have not been reported. To this end, this study used HS-SPME-GC-MS (headspace solid phase microextraction-gas chromatography-mass spectrometry) to measure the flavor substances in southern stinky tofu brine. Then, we carried out systematical optimization of factors such as chromatographic column, extraction fiber, equilibrium time, extraction temperature, and extraction time. After this, we analyzed the differences and similarities of flavor substances in different brands of brine by principal component analysis (PCA) for the purpose of providing a theoretical basis for flavor analysis, the formation mechanism, quality standard establishment, and processing of stinky tofu in the future.

## 2. Results and Discussion

### 2.1. Selection of Chromatographic Column

**Figure 1:**
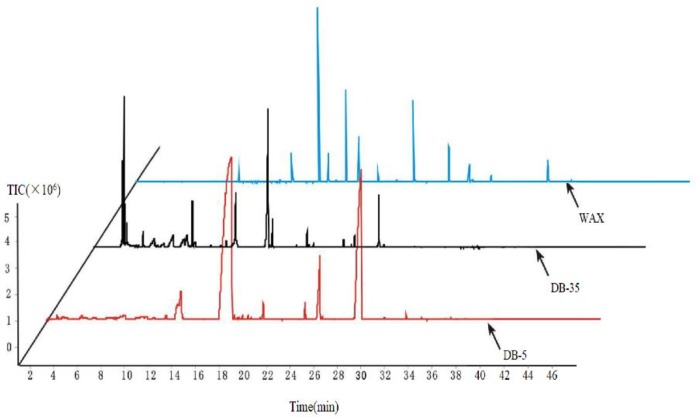
Total ion chromatogram by different chromatographic columns.

**Figure 2:**
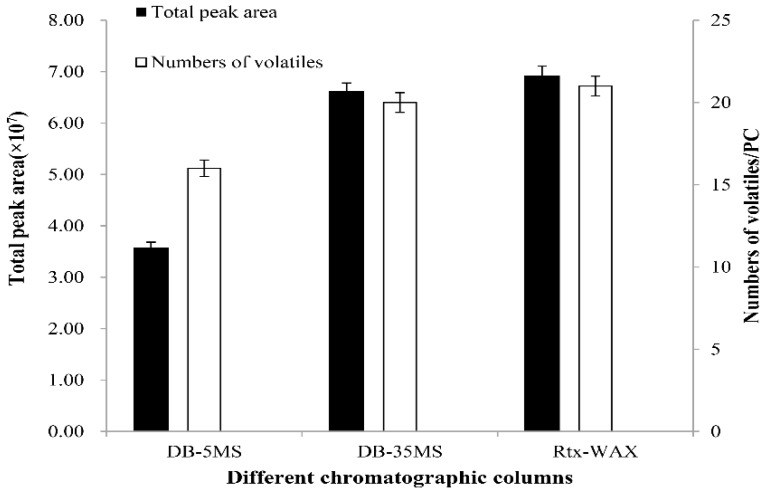
Effect of different chromatographic columns on total peak area and numbers of volatiles from stink tofu brine.

In this experiment, the volatile components of stinky tofu brine were separated by three chromatographic columns of different degrees of polarity. The total ion flow chart, the effective peak number and the total peak area obtained are shown in Figure 1 and Figure 2. As shown in Figure 1, the chromatographic peaks obtained by non-polar column DB-5MS are too wide, and serrated spectral peaks appear at a retention time of 6–15 min. The chromatographic peaks obtained by the middle-polar column DB-35MS are regular and serrated spectral peaks also appear at the retention time of 10–15 min. The chromatographic peaks obtained by the polar column CD-WAX are standard gauss spectral peaks. These show a clear and regular distribution with less overlapping interference. As shown in Figure 2, the total peak area and the effective peak number of volatile components detected by non-polar column DB-5MS are the least. The total peak area and the effective peak number of volatile components detected by the middle polar column DB-35MS and the polar column CD-WAX are similar to each other. The polar CD-WAX chromatographic column can separate out indole—a special volatile compound. In conclusion, the polar chromatographic column CD-WAX is the optimum chromatographic column for the separation purpose.

### 2.2. HS-SPME Condition Optimization

#### 2.2.1. Selection of Extraction Fiber

The number of active volatile substances in brine detected by seven different types of extraction fibers can be sorted as shown in Table 1: pink (22) > black (19) > red (19) > white (18) > blue (15) > orchid (13) > grey (10). The difference among white, pink, black, and red is not significant (*p* > 0.05). Total peak area can be ranked as: white (12.97 × 10^7^) > pink (8.79 × 10^7^) > black (6.92 × 10^7^) > blue (3.58 × 10^7^) > red (3.01 × 10^7^) = orchid (3.01 × 10^7^) > grey (0.35 × 10^7^) and there is significant difference between white, pink, and black extraction fibers (*p* < 0.05). Through further analysis of pink and white extraction fibers which have better extraction results, it can be shown that the effective peak number of volatile matter obtained by pink fiber extraction is higher, but not significantly different from that obtained by white fiber extraction (*p* > 0.05). The total peak area of volatile matter obtained by the white extraction fiber is the highest (12.97 × 10^7^). This is significantly different from that obtained by other extraction fibers (*p* < 0.05). This indicates that white extraction fiber has the best loading capacity and can detect alcohol. Therefore, the white extraction fiber was adopted in this study. It has been reported that Liu studied the extraction conditions of volatile components of northern stinky tofu and found that the black extraction fiber 75 μm Carboxen/PDMS (Polydimethylsiloxane) was the most suitable for extraction [7]. In this experiment, the black extraction fiber had a good extraction effect on alcohol, aldehyde, ketone, ether, heterocyclics, and other substances, but the total peak area of volatile substances extracted was relatively low. This may be due to the raw materials of stinky tofu in the north and south forms being different. The alternative extraction fibers were also different. In Liu’s work, only four extractive fibers were selected (not white), so the results were different to ours. Therefore, the white 85 μm Polyacrylate extraction fiber was considered as the optimal extraction fiber in this experiment.

#### 2.2.2. Selection of Extraction Temperature

**Figure 3:**
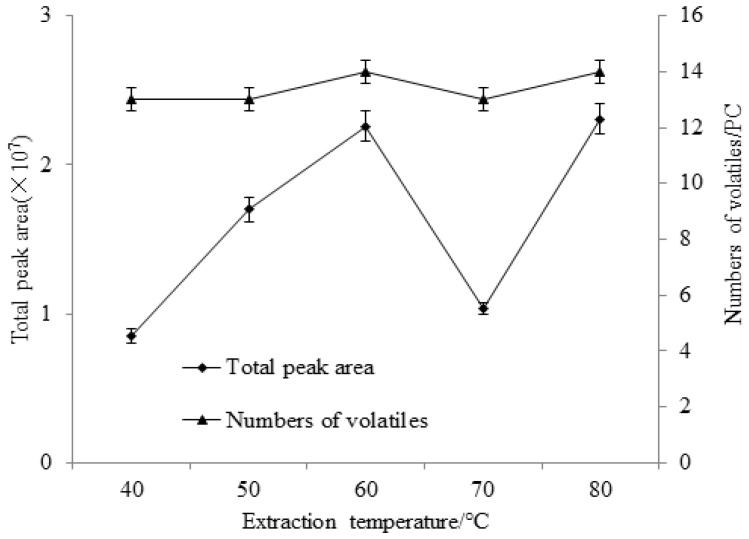
Effect of extraction temperature on total peak area and numbers of volatiles from stink tofu brine.

The influences of extraction temperature on the total peak area and the effective peak number of volatile flavor substances in stinky tofu brine are shown in Figure 3. The extraction temperature has a certain influence on the extraction effect of volatile components of brine. When the extracting temperature is between 40–80 °C, the total peak area and effective peak number of the volatile components obtained vary greatly; when the extraction temperature is 60 °C or 80 °C, the effective peak number and the total peak area are both high and there is no significant difference between the two (*p* < 0.05). However, an excessively high extraction temperature may lead to decomposition of some volatile components, making the volatile components in brine not being reduced well so the optimal extraction temperature was set to 60 °C. The diffusion and partition coefficients of the SPME adsorption process are different for each substance at different temperatures. The total peak area decreased at 70 °C may be mainly due to the two-sided effect of the extraction temperature on the SPME method [10,11]. On the one hand, under kinetic control, increasing the temperature (40–60 °C) accelerates the thermal motion of the molecules, facilitates the diffusion of the analytes in the substrates (increases the distribution of the analytes in the headspace), shortens the equilibration time, and hence speeds up the analysis. On the other hand, under thermodynamic control, since the SPME extraction and adsorption process is an exothermic process, increasing the temperature (60–70 °C) will reduce the partition coefficient of the analytes in the substrates, resulting in a decrease in the adsorption of the analytes on the coating. At higher temperatures (70–80 °C), the sample molecules move more vigorously, and the intensity of the molecular movement is much greater than the distribution coefficient, leading to a rise in the total peak area.

#### 2.2.3. Selection of Equilibrium Time

**Figure 4:**
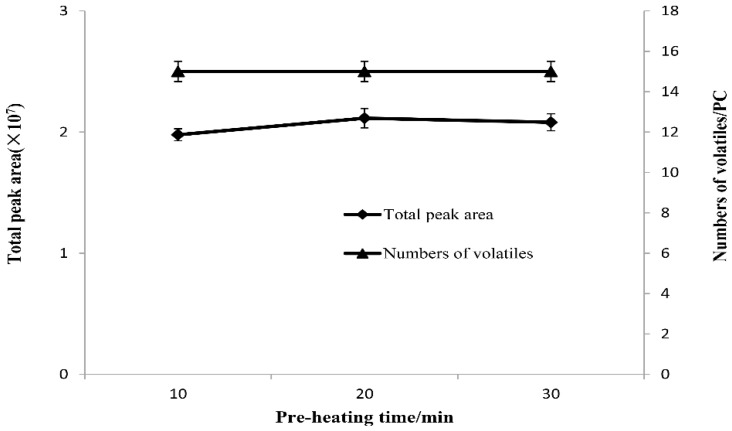
Effect of pre heating time on total peak area and numbers of volatiles from stink tofu brine.

The main function of equilibrium is to release sufficient volatile components from the sample. The effects of equilibrium time on the total peak area and the effective peak number of volatile flavor substances in the stinky tofu brine are shown in Figure 4. For the three equilibrium times, the number of effective peaks extracted by the extraction fiber is the same (15), but the total peak area varies to a certain extent. When the equilibrium time is 20 min, the peak area is the largest (2.11 × 10^7^); as the equilibrium time increases to 30 min, the peak area changes insignificantly (2.08 × 10^7^). Therefore, the optimal equilibrium time was set at 20 min.

#### 2.2.4. Selection of Extraction Time

**Figure 5:**
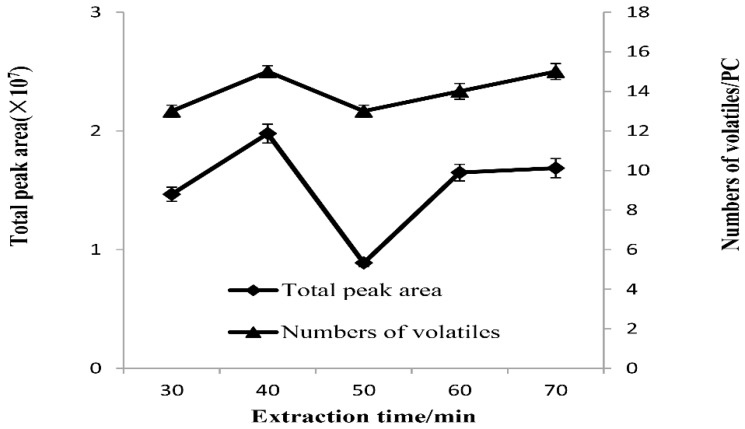
Effect of extraction time on total peak area and numbers of volatiles from stink tofu brine.

The influences of extraction time on the total peak area and effective peak number of volatile flavor substances in stinky tofu brine are shown in Figure 5. For all five extraction times selected, the effective peak number and total peak area of volatile matter obtained by extraction fiber showed a trend of increasing first, then decreasing, and increasing again. The effective peak number and total peak area of extracted fiber were higher at extraction times of 40 min and 70 min. Extraction is a dynamic equilibrium process of adsorption and resolution. Within a certain period of time, the species and amount of substances adsorbed on the extraction fiber increased with the extension of time. With the further extension of time, the content of some extracted substance increased, making the absorption of extraction fiber become saturated, although some volatile substances were not strongly adsorbed but were dissolved from the extraction fiber. This resulted in the total number of odor components decreasing. For example, at an extraction time of 30 min, the adsorption and analysis of volatile components on the extraction fibers did not reach equilibrium while at an extraction time of 40 min, adsorption and resolution basically reached equilibrium. When the extraction time reached 70 min, the adsorption and analysis reached a new equilibrium again. However, the total peak area and effective peak number of volatile matter retained on the extracted fiber decreased significantly. This is consistent with the reported results in the literature [7,10]. Therefore, the optimal extraction time in this experiment was set to 40 min.

### 2.3. Results and Analysis of Volatile Components in Five Brands of Stinky Tofu Brine

#### 2.3.1. GC-MS Results of Volatile Components in Five Brands of Stinky Tofu Brine

The volatile components in five brands of brine were determined under the optimal SPME-GC-MS condition, as shown in Table 2. A total of 63 volatile flavor compounds were identified in five brands of brine. There were 29, 30, 21, 45, and 16 volatile flavor compounds in Chengshifu brine (STB1), (Huogongdian brine) STB2, Wangcheng brine (STB3), Baise jingdian brine (STB4), and Luojia brine (STB5), respectively. This accounts for 99.06%, 99.76%, 78.39%, 98.76%, and 78.85% of the total volatile matter, respectively. Of the five brands of brine, the relative contents of acids (52.29%), phenols (23.13%), and indoles (22.99%) are higher in STB1. In STB2, phenols (47.76%) and indoles (35.28%) are predominant, and the relative contents of ester (5.6%), alcohol (4.83%), and acid (4.39%) are also higher. In STB3, the relative contents of various substances are relatively uniform, and the relative contents of alcohols, acids, esters, phenols, ether, and indoles are 12.15%, 12.32%, 23.23%, 10.57%, 14.97%, and 5.15%, respectively. In STB4, acids (44.52%), phenols (31.70%), and indoles (17.52%) are the main volatile components. In STB5, the relative contents of esters (41.72%) and phenols (20.56%) are higher. The same results show acids, sulfur-containing compounds and indoles accounting for 5.84%, 7.3%, and 3.48%, respectively. In conclusion, phenols are the main components of the volatile substances in the five kinds of brine. Acids and indoles are also important characteristic flavor components in the five kinds of brine. Esters are the main flavor components in STB3 and STB5.

A total of six phenols were detected from five brines (3, 4, 4, 4, and 3 phenols in STB1, STB2, STB3, STB4, STB5, respectively). Although there were not many different types, they were relatively high in content and the main components of volatile substances in all five brines. By analyzing the phenolic compounds in five brines, it was found that p-methylphenol and phenol both existed in the five brine samples. The content of p-methylphenol was the highest, which was 2.75–43.88%. Para-methylphenol has a special odor. Studies have shown that the odor of bamboo shoots fermented by traditional techniques in Taiwan is caused by para-methylphenol [12,13,14]. Tyrosine is the main free amino acid in the tender part of bamboo shoots, which can be decomposed into para-methylphenol. Para-methylphenol and skatol (i.e., 3-methylindole) together constitute pig skatole. The raw material of stinky tofu brine contains bamboo shoots, so it is speculated that p-methylphenol may come from bamboo shoot fermentation products. The relative content of phenol in five brines was 0.97–3.72%. Phenol has a phenolic odor of plastic and rubber, which enhances the odor of the brine. The structural formula of tyrosine contains phenol. Therefore, it is speculated that phenol may come from the decomposition of tyrosine, and there may be some correlation between phenol and p-methylphenol [14,15,16,17]. Liu found that phenol was also the main characteristic aroma component of Wangzhihe stinky tofu, which is consistent with the results of this study [7].

A total of 16 acids were detected from five brines, including 14, 10, 7, 13, and 5 acids in STB1, STB2, STB3, STB4, and STB5, respectively. The acids shared by the five brines are acetic acid, propionic acid, isobutyric acid, butyric acid, and 3-methylbutyric acid (specific relative contents are shown in Table 2). Volatile fatty acids are the main end product of intestinal metabolism of bacteria in the large intestine. These are mainly produced by the fermentation of indigestible carbohydrates by anaerobic microorganisms [18]. Acetic acid has a strong pungent and sour taste. Propionic acid, isobutyric acid, and 3-methylbutyric acid have a certain pungent smell, with a sour and rancid taste. Butyric acid has a rancid, buttery, and cheesy aroma [19,20].

Two kinds of indoles (3-methylindole and indole) were detected from the five kinds of brine, and the 3-methylindole content was relatively higher, which was 2.78–33.68%. 3-methylindole, also known as skatole, has a special unpleasant flavor, with an extremely low threshold. 3-methylindole and 4-methylphenol may be the degradation products of tryptophan [15]. The indole, with relative content of 0.70–2.60%, has a strong unpleasant special flavor, with a low threshold (140 µg/L^6^). Liu found that 3-methylindole and indole were the characteristic flavor substances in brine, which is consistent with the results of this paper [7].

In addition, it can be seen from Table 2 that the contents of esters (mainly n-hydroxy-benzoicacimethylester) in STB3 and STB5 are up to 23.23% and 41.72%, respectively. However, the flavor of such substances is still unclear [21].

In conclusion, the “offensive odor” of stinky tofu is mainly from phenols, volatile fatty acids, and indoles. Among them, p-methylphenol, phenol, 3-methylindole, indole, acetic acid, propionic acid, isobutyric acid, butyric acid, and 3-methyl-butyric acid are the common contributing odors in the substances for all five kinds of brine.

#### 2.3.2. Principal Component Analysis Results of Volatile Components in Five Brands of Stinky Tofu Brine

**Figure 6:**
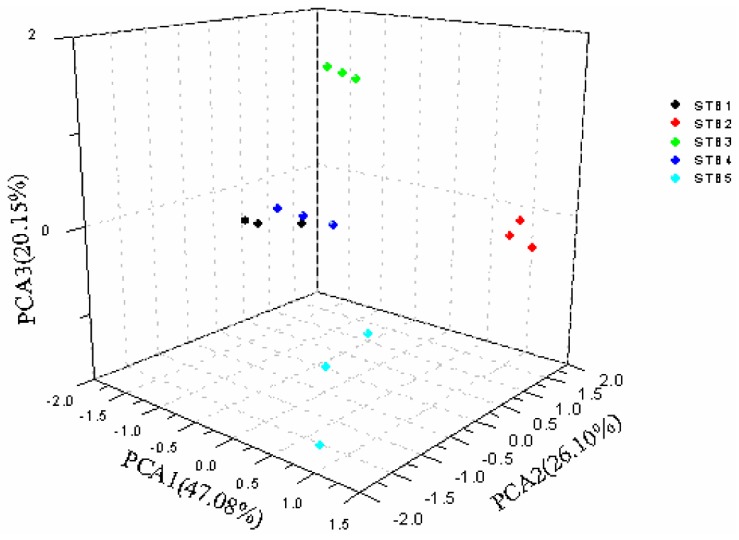
Principal component analysis of five brands of brine.

Volatile data obtained from SPME/GS-MS were elaborated by PCA to compare the profile of volatile compounds using SPSS 19.0 and Origin 8.0 software (Table 3 and Figure 6). ANOVA was applied to select the most discriminant compounds in order to improve separation among samples. As shown in the figure, the score plot revealed that principal components (PCs) PC1 (acids, aldehydes, ketones, and heterocyclic compounds), PC2 (indoles, phenols, ethers, and sulfur compounds), and PC3 (sulfur compounds, alcohols, and ethers) represented 47.8%, 26.10%, and 20.15% of the total variance, respectively.

The cumulative contribution rate of the first three PCs was 93.33%, which seem to provide enough information on the stinky tofu samples.

As seen in Figure 3, STB1 and STB4 are relatively similar in PC1, 2 and 3, indicating these two brines have a similar flavor. Therefore, we speculate that they are similar in the formulation and production process. STB2 and STB3 can be classified into the same group (PC3) based on the PCA results. STB5 significantly differs from other brands of brine, thus showing a significantly different flavor. The flavor of brine determines the quality and characteristics of the stinky tofu. Results obtained from this study suggest that the analysis of the difference in the brine flavor can provide a good theoretical basis for the flavor characterization and the processing of stinky tofu in the future.

## 3. Materials and Methods

### 3.1. Materials and Reagents

Five kinds of stinky tofu brine were collected from different brine enterprises in Changsha in Hunan province made by natural fermentation of tofu, black bean sauce, mushrooms, bamboo shoots, mustard or dried plum for one year. These are denoted as STB1, STB2, STB3, STB4, and STB5 respectively.

### 3.2. Instruments and Equipment

Instruments include GC-MS-QP2010 gas chromatograph-mass spectrometer (Shimadzu, Japan), B-5MS weak-polar chromatographic column, DB-35MS medium-polar chromatographic column, CD-WAX polar chromatographic column, SPME sample injector, SPEM holder, 15 mL headspace sampling bottle. All seven extraction fibers were purchased from Sueplco of the United States. Talboys digital display magnetic heating agitator and 12 × 4.5 mm PTFE octagon pivot magnet were purchased from Shanghai Anpu Scientific Instrument Co., Ltd. (Shanghai, China); other conventional instruments and equipment were provided by the Laboratory of Food Science and Technology College of Hunan Agricultural University.

### 3.3. Methods

#### 3.3.1. Selection of Chromatographic Column

By randomly selecting a brine (STB2) as the test sample, the analysis conditions of the separation column was optimized using the single-factor stepwise optimization method. The total ion flow chart, effective peak number, and total peak area of the sample were investigated using a DB-5MS weak polar analytical fused-silica capillary column (30 m × 0.25 mm × 0.25 μm, CNW, ANPEL, Shanghai, China), DB-35MS medium polar analytical fused-silica capillary column (60 m × 0.25 mm × 0.25 μm, CNW, ANPEL) and CD-WAX polar analytical fused-silica capillary column (30 m × 0.25 mm × 0.25 μm, CNW, ANPEL, Shanghai, China). Other analytical conditions were as follows: extraction heads black 75 μm Carboxen/PDMS, equilibrium time 20 min, extraction temperature 60 °C, extracting time 40 min. Analysis of the sample at each condition was repeated 3 times.

#### 3.3.2. Optimization of HS-SPME-GC-MS Analysis Condition

By randomly selecting a brine (STB2) as the test sample, the analysis condition of extraction head type, extraction temperature, equilibrium time and extraction time were optimized using single-factor stepwise optimization methods [10,22]. According to the differences in polarity and coating thickness, a total of seven extraction heads (Sueplco, Bellefonte, PA, USA) were employed. The fiber specifications are shown in Table 4, other analytical conditions were: CD-WAX chromatographic column, 20 min equilibrium time, 60 °C extracting temperature, 40 min extracting time, to investigate the volatile components, effective peak number and total peak area of sample. The total peak area and effective peak number were investigated by setting extracting temperature as 4 gradients (30 °C, 40 °C, 50 °C, and 60 °C, other analytical conditions were: CD-WAX chromatographic column, white 85 μm phenyl-m polyacrylate extraction heads, 20 min equilibrium time, 40 min extracting time). By setting equilibrium time as 3 gradients (10 min, 20 min, and 30 min, other analytical conditions were set as follows: CD-WAX chromatographic column, white 85 μm phenyl-m polyacrylate extraction heads, 60 °C extracting temperature, 40 min extracting time) the total peak area and effective peak number were investigated. By setting extracting time as 5 gradients (30 min, 40 min, 50 min, 60 min, and 70 min, other analytical conditions were as follows: CD-WAX chromatographic column, white 85 μm phenyl-m polyacrylate extraction heads, 60 °C extracting temperature, 20 min equilibrium time). The total peak area and effective peak number were analyzed and the analysis was repeated three times for each sample. The optimum extraction and analysis conditions of HS-SPME-GC-MS were determined according to the change of the corresponding indexes under different gradients.

#### 3.3.3. Extraction of Volatile Components.

The volatile components of stinky tofu were extracted by the HS-SPME method. When the extraction fiber was first used, it was aged according to the conditions recommended by the manufacturer until no interference peak appeared on the chromatographic detection chart. Stinky tofu brine (5 mL) was taken with a pipette and sealed in a 15 mL top-empty bottle. Then, the extraction was carried out on the SPME device which consists of a 15-mL vial puck, a PK1 holder, a Talboys 7 × 7 hotplate-stirrer, and vials (15 mL) under different extraction conditions, with the rotate speed set to 200 r/min, and then subjected to analysis at the GC injection port at 240 °C for 5 min.

#### 3.3.4. GC-MS Measurement Condition

The GC (QP2010, Shimadzu, Japan) condition: the carrier gas is helium (99.999%), with flow velocity of 1.0 mL/min; the temperature at injection port is 240 °C; splitless injection was adopted. Temperature program: column temperature was at 50 °C, holding for 2 min, heated to 200 °C by 5 °C/min, holding for 14 min and then heated to 240 °C by 15 °C/min. The MS (QP2010, Shimadzu, Japan) condition: the ion source is Electron Ionization (El), with ion source temperature of 200 °C; electron energy 70 eV; emission current 150 μA; multiplier voltage i 1037 V; interface temperature 220 °C; mass scan range 45–500 *m*/*z*.

#### 3.3.5. Data Analysis

Qualitative analysis: the total ion flow chromatogram was retrieved from the NIST2017s (Standard Spectrum Library of the National Institute of Standards and Technology of the United States). The compound was determined by calculating the retention index [23]. The retention index was calculated by formula (1):

(1) $$I=100\times \left(n+\frac{t_{i}-t_{n}}{t_{n+1}-t_{n}}\right)$$

where I = the retention index; n = carbon number; t_i_ = the retention time for volatile components to be measured; t_n_ = the retention time for n-carbon normal alkane; t_n+1_ = the retention time for (n + 1)-carbon normal alkane.

Quantitative analysis: the area normalization method was used for relative content analysis using the following formula (2):

Mi% = Ai/∑Ai × 100%(2)

where Mi is the percentage of the measured component i; Ai is the peak area of the measured component i; ∑Ai is the total peak area.

Single-factor ANOVA analysis and PCA were performed using SPSS 19.0 and Origin 8.0 software.

## 4. Conclusions

Based on the results of the HS-SPME-GC-MS condition optimization tests, the optimal conditions for analysis of volatile components in stinky tofu brine were obtained as follows: DB-WAX chromatographic column; white 85 microns polyacrylate extraction fiber, extraction temperature of 60 °C; 20 min as the equilibrium time, and 40 min for the extraction time.

HS-SPME-GC-MS detection was carried out for the five brands of brine under superior analysis conditions and 63 volatile flavor substances were identified. This includes 29, 30, 21, 45, and 16 substances for STB1, STB2, STB3, STB4, and STB5, respectively, accounting for 99.06%, 99.76%, 78.39%, 98.76%, and 78.85% of the total volatile substances. These are mainly alcohols, acids, esters, aldosterone, phenols, ether, sulfur-containing compounds, indoles, heterocycle, etc. Phenols are the main component of the five kinds of brine. Acids and indoles are the most important characteristic flavor components. Esters are the main flavor components in STB3 and STB5. Para-methylphenol, phenol, 3-methylindole, indole, acetic acid, propionic acid, isobutyric acid, butyric acid, and 3-methyl-butyric acid are the common odorous contributing substances shared by the five kinds of brine and are also the source of the “offensive odor” of stinky tofu.

Different brands of brine have big differences in flavor. Through PCA analysis, the cumulative contribution rate of the first three PCs was 93.33%, which appeared to provide enough information on the stinky tofu samples. It was found that STB1 and STB4 were relatively similar in PC 1, 2, and 3 and the two brines had similar flavor. Therefore, it was speculated that they were similar in formula and production process. STB2 and STB3 can be classified into the same group (PC 1), but there is a big difference in other PCs for the two brines. STB5 significantly differed from other brands of brine in the PC 1–3, showing significantly different flavors. Results obtained from this study indicated that determining the volatile substances of brine could be used as an indicator for the flavor characterization and the processing of stinky tofu.

Although the relative contents of volatile components in different brands of brine have been analyzed and compared, the difference in flavor of different brands of brine was obtained in this study. However the active flavor of each brine was not determined. Therefore, future work will focus on determination of the main active flavor substances in brine using internal standard quantification, threshold comparison or GC-O-MS methods. This will provide a theoretical basis for the establishment of brine quality standards, quality control, and large-scale industrial production of stinky tofu in future studies.

## Figures and Tables

**Table 1:** Results of the volatile components from seven kinds of SPME extraction.

Numbers of Volatiles	White	Pink	Black	Red	Grey	Blue	Orchid
Acids	10	10	6	12	6	10	7
Phenols	3	3	3	2	2	3	3
Alcohols	1	-	1	-	-	-	-
Esters	1	1	-	-	-	-	-
Aldehydes and ketones	1	5	4	4	-	-	-
Sulfur compounds	-	-	2	-	-	-	-
Alkenes	-	-	-	1	-	-	-
Heterocyclic compounds	2	3	4	2	2	2	3
Numbers of volatiles/PC	18 ± 2.0 ^abc^	22 ± 2.4 ^a^	19 ± 2.0 ^abc^	19 ± 2.0 ^ab^	10 ± 0.8 ^e^	15 ± 1.2 ^bcd^	13 ± 1.6 ^de^
Total peak area (×10^7^)	12.97 ± 1.01 ^a^	8.79 ± 0.87 ^b^	6.92 ± 0.81 ^c^	3.01 ± 0.55 ^def^	0.35 ± 0.10 ^g^	3.58 ± 0.62 ^d^	3.01 ± 0.60 ^de^

-: signifies undetected; the same alphabetical letter in a column signifies the difference is not significant at the level of *p* = 0.05, and different alphabetical letters signify the difference is significant.

**Table 2:** Relative contents and flavor characteristics of volatile compounds in five stink tofu brines.

No.	Compounds Name	RI ^a^	RI ^b^	CAS	Relative Contents/% (of the Total Area)					Odor
					STB1	STB2	STB3	STB4	STB5	
**Alcohols**					0.27 ± 0.09	4.83 ± 1.01	12.15 ± 2.55	3.25 ± 0.23	0.73 ± 0.15	
1	Ethanol	**N/A ^c^**	934–937	64-17-5	0.04 ± 0.01	1.30 ± 0.20	3.53 ± 1.46	0.13 ± 0.06	0.73 ± 0.15	Bouquet, alcohol
2	Isoamyl alcohol	1254	1233–1268	71-41-0	- ^d^	-	-	0.28 ± 0.01	-	Floral, fruity
3	Hexanol	1322	1354	111-27-3	-	-	-	0.24 ± 0.01	-	Floral, fruity
4	Octan-1-ol	1535	1539–1558	111-87-5	-	-	-	0.03 ± 0.01	-	Floral, fruity, citrus
5	4-terpineol	1573	15581	562-74-3	-	-	-	0.02 ± 0.01	-	Peppery
6	1,5-hexanediol	1597	N/B ^e^	142-68-7	-	1.58 ± 0.38	-	-	-	N/O ^f^
7	Phenylethyl alcohol	1871.79	1875–1899	60-12-8	0.02 ± 0.01	-	-	0.16 ± 0.01	-	Slightly roses
8	1,4-Butanediol	1877.53	1861	110-63-4	0.06 ± 0.02	0.27 ± 0.08	1.99 ± 0.88	0.25 ± 0.08	-	N/O
9	Diethylene glycol	1926	1953	111-46-6	-	-	0.64 ± 0.06	-	-	Spicy
10	3-Phenylpropan-1-ol	2003	2022	122-97-4	-	-	-	0.10 ± 0.01	-	Floral, fruity
11	1-Tetradecanol	2139	2152–2172	112-72-1	-	-	-	0.03 ± 0.01	-	Waxy
12	4-(Isopropylamino)butanol	2439	N/B	31600-69-8	0.15 ± 0.05	1.68 ± 0.39	5.99 ± 2.64	2.01 ± 0.70	-	N/O
**Acids**					52.29 ± 7.43	4.39 ± 1.64	12.32 ± 2.52	44.52 ± 3.87	5.84 ± 1.38	
1	Acetic acid	1425.65	1424–1446	64-19-7	15.39 ± 3.41	0.60 ± 0.25	3.78 ± 0.64	3.47 ± 0.26	1.52 ± 0.34	Pungency, sour, acidic
2	Propionic acid	1510.13	1490–1525	79-09-4	10.77 ± 1.29	0.27 ± 0.08	1.34 ± 0.94	4.43 ± 0.33	1.01 ± 0.03	Pungency
3	Isobutyric acid	1535.36	1524–1561	79-31-2	8.27 ± 0.24	0.44 ± 0.17	0.64 ± 0.12	1.41 ± 0.07	0.08 ± 0.02	Rancid, sweat
4	n-Butyric acid	1593.14	1593–1607	107-92-6	2.54 ± 1.82	0.63 ± 0.54	4.63 ± 0.44	10.46 ± 0.63	3.12 ± 0.94	Fatty, rancid
5	3-Methylbutanoic acid	1637.15	1633–1643	503-74-2	5.60 ± 0.99	1.86 ± 0.56	1.43 ± 0.27	7.15 ± 0.45	0.44 ± 0.13	Cheese, rancid
6.	Valeric acid	1703.40	1698–1730	109-52-4	5.19 ± 0.38	0.23 ± 0.02	0.33 ± 0.13	10.79 ± 0.67	-	Cheese, sweat
7	2-Methylpentanoic acid	1733.97	1746	97-61-0	0.51 ± 0.08	-		-	-	Pungent, spicy,
8	4-Methylpentanoic acid	1768.09	1774–1809	646-07-1	1.67 ± 0.28	0.07 ± 0.03	0.17 ± 0.12	0.51 ± 0.05	-	Acidic, rancid
9	1-Hexanoic acid	1808.81	1802–1812	142-62-1	1.25 ± 0.09	0.20 ± 0.07	-	5.28 ± 0.21	-	Rancid
10	2-Methylhexanoic acid	1831.54	N/B	4536-23-6	0.90 ± 0.07	0.08 ± 0.03	-	0.02 ± 0.01	-	Rancid
11	3-Methylhexanoic acid	1866.26	1869	3780-58-3	0.04 ± 0.01					Acidic, foul
12	n-Heptanoic acid	1909.262	1900–1923	111-14-8	0.03 ± 0.01	0.01 ± 0.01	-	0.69 ± 0.01	-	Acidic
13	Octanoic acid	2017.68	2011–2075	124-07-2	0.11 ± 0.01	-	-	0.25 ± 0.03	-	Acidic, sweat
14	Decanoic acid	2231	2237–2244	334-48-5	-	-	-	0.03 ± 0.01	-	Acidic
15	Phenylacetic acid	2517	2528	103-82-2	-	-	-	0.03 ± 0.01	-	Honey
16	3-Phenylpropanoic acid	2573.83	2603	501-52-0	0.02 ± 0.01	-	-	-	-	Slightly sweet
**Esters**					0.03 ± 0.02	5.60 ± 0.17	23.23 ± 6.76	1.21 ± 0.41	41.72 ± 8.71	
1	Dimethyl sulfite	1219	N/B	616-42-2	-	0.01 ± 0.01	-	-	-	Onion
2	4-Hydroxybutyric acid	1599	N/B	591-81-1	-	-	-	0.07 ± 0.02	-	N/O
3	Methyl N-hydroxybenzenecarboximidoate	1732	N/B	-	-	4.85 ± 0.20	21.09 ± 8.01	0.11 ± 0.04	41.72 ± 8.71	N/O
4	2-Ethoxyethyl acrylate	1953	N/B	106-74-1	-	0.03 ± 0.02	-	0.39 ± 0.01	-	N/O
5	4-Hydroxybutyl acrylate	2325	N/B	2478-10-6	0.03 ± 0.02	0.47 ± 0.36	2.14 ± 1.03	0.64 ± 0.21	-	N/O
6	2-Isocyanato-1,3-dimethylbenzene	2718	N/B	28556-81-2	-	0.24 ± 0.08	-	-	-	Floral, fruity
**Aldehydes and ketones**					0.25 ± 0.05	0.03 ± 0.01	-	0.17 ± 0.06	-	
1	2,4,5-Trimethylbenzaldehyde	1823	1896	5779-72-6	-	0.01 ± 0.01	-	0.02 ± 0.01	-	N/O
2	2-Pentadecanone	1986	1998-2023	2345-28-0	-	-	-	0.02 ± 0.01	-	No flavor
3	Apricolin	1980	1990	104-61-0	-	-	-	0.03 ± 0.01	-	Fruity, coconut
4	2-Aminoacetophenone	2144	2187	551-93-9	-	-	-	0.07 ± 0.02	-	Pungent
5	2-Piperazinone	2071.24	2060	675-20-7	0.24 ± 0.07	-	-	0.03 ± 0.01	-	N/O
6	Heptadecan-2-one	2193.21	2196	2922-51-2	0.01 ± 0.01	0.02 ± 0.01	-	-	-	N/O
**Phenols**					**23.13 ± 2.35**	**47.76 ± 3.33**	**10.57 ± 1.25**	**31.70 ± 4.12**	**20.56 ± 3.92**	
1	Phenol	1959.29	1950-1989	108-95-2	1.93 ± 0.25	2.64 ± 0.28	0.97 ± 0.34	2.75 ± 0.08	3.72 ± 0.69	Rubbery, skunk, phenolic
2	p-Cresol	2035.59	2050	106-44-5	21.04 ± 2.11	43.88 ± 2.95	2.75 ± 0.45	19.29 ± 4.09	16.84 ± 3.28	Phenolic, stink
3	m-Cresol	2054	2059	108-39-4	-	-	-	0.48 ± 0.03	-	Phenolic, stink
4	4-Ethylphenol	2125.58	2167-2175	123-07-9	0.16 ± 0.03	1.23 ± 0.05	2.52 ± 0.59	9.14 ± 0.03	-	Spicy, rancid
5	4-Propylphenol	2211	2234	645-56-7	-	-	4.33 ± 0.99	0.04 ± 0.01	-	N/O
6	4-Isopropenylphenol	2397	N/B	4286-23-1	-	0.01 ± 0.01	-	-	-	Leather, skunk, rancid
**Ethers**					0.01 ± 0.01	0.95 ± 0.14	14.97 ± 4.26	0.14 ± 0.05	0.12 ± 0.03	
1	Dimethyl ether	N/A	478	115-10-6	-	-		0.13 ± 0.04	-	Ether
2	2-Ethoxyethanol	1246	1239-1246	110-80-5	0.01 ± 0.01	0.04 ± 0.02	0.78 ± 0.23	0.01 ± 0.01	0.12 ± 0.03	Stink
3	2-(2-Ethoxyethoxy) ethanol	1595	1622	111-90-0	-	0.91 ± 0.13	14.19 ± 3.98	-	-	Stink
**Sulfur compounds**					-	-	-	-	7.3 ± 2.19	
1	Sulfur Dioxide	N/A	882	7446-09-5	-	-	-	-	7.3 ± 2.19	Pungent
**Alkanes**					-	-	-	0.04 ± 0.01	-	
1	α-Curcumene	1734	1738-1786	644-30-4	-	-	-	0.04 ± 0.01	-	N/O
**Indoles**					22.99 ± 2.80	35.28 ± 2.80	5.15 ± 0.45	17.52 ± 1.66	3.48 ± 0.87	
1	Indole	2375.14	2412	120-72-9	2.60 ± 0.47	1.60 ± 0.06	1.23 ± 0.24	0.79 ± 0.05	0.70 ± 0.15	Stool, rancid
2	3-Methylindole	2420.30	2459	83-34-1	20.39 ± 2.38	33.68 ± 3.42	3.92 ± 0.28	16.73 ± 1.06	2.78 ± 0.72	Stool, stink, rancid
**Heterocyclic compounds and others**					0.09 ± 0.02	0.92 ± 0.46	-	0.21 ± 0.05	2.47 ± 0.66	
1	Trimethylamine	N/A	546	75-50-3	-	-	-	0.06 ± 0.02	-	Fish oil
2	Urea	N/A	N/B	57-13-6	-	-	-	0.09 ± 0.03	-	Odorless
3	1,2,4-Trithiolane	1704	1760	289-16-7	-	-	-	-	2.09 ± 0.57	N/O
4	Formamide	1742.60	1772	75-12-7	0.05 ± 0.01					Slight ammonia
5	Butanamide	1848.27	1901	541-35-5	0.04 ± 0.03	-	-	-	-	N/O
7	1,3,3-Trimethyl-1-phenylindan	2171	N/B	3910-35-8	-	-	-	-	0.24 ± 0.05	N/O
8	(2,3-dimethyl-3-phenylbutan-2-yl) benzene	2319	N/B	1889-67-4	-	-	-	-	0.14 ± 0.04	N/O
9	4-Pyrrolidinopyridine	2324	N/B	2456-81-7	-	-	-	0.06 ± 0.02	-	N/O
10	Methanesulfonyl chloride	1203	1216	124-63-0	-	0.92 ± 0.46	-	-	-	N/O
Total					99.06 ± 1.31	99.76 ± 0.31	78.39 ± 5.31	98.76 ± 1.11	78.85 ± 6.20	

^a^ Retention indices calculated on DB-WAX column against n-alkanes; ^b^ Retention indices reported by http://webbook.nist.gov/chemistry/cas-ser.html; ^c^ not calculated; ^d^ not detected; ^e^ Data not available; ^f^ The flavor is not clear.

**Table 3:** Rotational component matrix.

Compounds	Component		
	1	2	3
Acids/%	−0.980		
Aldehydes and ketones/%	−0.942		
Heterocyclic compounds and others/%	−0.937		
Indoles/%		0.956	
Phenols/%		0.817	
Ethers/%		−0.780	
Sulfur compounds/%		−0.642	−0.609
Alcohols/%			0.917
Ethers/%			0.899

**Table 4:** Parameters of SPME head with different specifications.

No.	Materials	Color	Coating Thickness/μm	Polarity
1	Polyacrylate	White	85	Polar
2	PDMS/DVB	Pink	65	Medium polar
3	PDMS	Red	100	Nonpolar
4	DVB/CAR/PDMS	Grey	50/30	Medium polar
5	Carboxen/PDMS	Black	75	Medium polar
6	Carboxen/PDMS	Blue	85	Medium polar
7	Carboxen/PDMS	Orchid	60	Medium polar

DVB: Divinylbenzene; CAR: Carboxen.
